# The main radiologic findings in annular pancreas

**DOI:** 10.1590/0100-3984.2017.0196

**Published:** 2019

**Authors:** Elazir B. M. Di Piglia, Claudia Renata R. Penna, Jeferson Tobias, Desirée Oliveira, Edson Marchiori

**Affiliations:** 1 Universidade Federal do Rio de Janeiro (UFRJ), Rio de Janeiro, RJ, Brazil.

Dear Editor,

A female infant was born at term without complications. At 12 days of life, she presented to a pediatric emergency department for investigation of frequent postprandial vomiting, weight loss, and irritability. According to the mother, she was eliminating urine and feces. Physical examination revealed abdominal distention. The laboratory findings were consistent with iron-deficiency anemia. An X-ray of the abdomen showed gaseous distention of the stomach and proximal duodenum, without gas in the distal portion, characterizing the typical double-bubble sign (Figure 1A). The findings were suggestive of duodenal obstruction. Abdominal ultrasound confirmed the X-ray findings, revealing distention of the stomach and duodenum. In addition, the ultrasound showed tissue surrounding the duodenum, suggesting a diagnosis of annular pancreas as the cause of the duodenal obstruction (Figures 1B and 1C). The patient underwent exploratory laparotomy, during which the diagnosis of duodenal obstruction caused by an annular pancreas was confirmed (Figure 1D). A diamond-shaped duodenoduodenostomy was performed, and the postoperative evolution was favorable.

**Figure 1:**
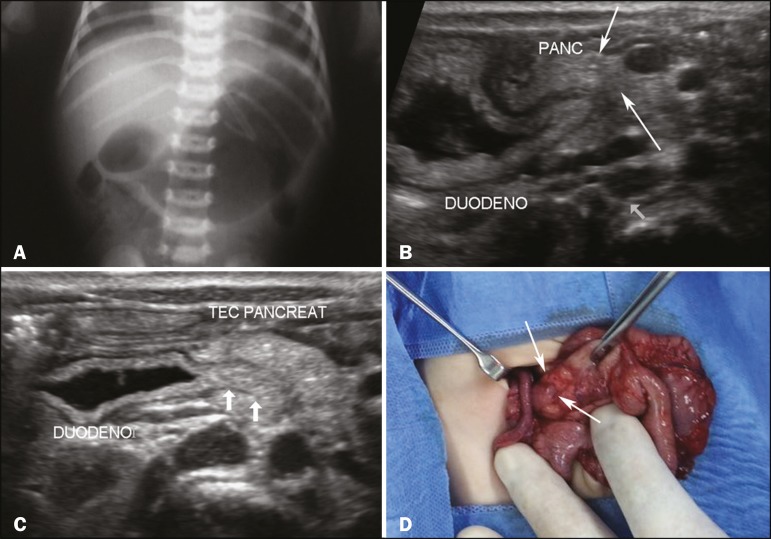
**A:** X-ray of the abdomen, showing gas distention of the stomach and duodenum, with little gas seen distally, characterizing the double-bubble sign. **B,C:** Ultrasound of the abdomen, showing pancreatic tissue (arrowheads in **B**) partially surrounding the duodenum (arrows in **C**). **D:** Photograph, obtained during laparotomy, confirming the presence of the pancreatic tissue (arrows) surrounding the duodenum.

Acute abdominal conditions are the subject of a number of recent studies in the radiology literature^(^^1^^-^^4^^)^. Congenital duodenal obstruction is relatively common during the neonatal period. It can be categorized as complete or partial and as intrinsic or extrinsic. Extrinsic duodenal obstruction has many causes, including annular pancreas, malrotation, and anterior portal vein^(^^5^^)^.

Annular pancreas is a rare congenital malformation, characterized by the development of a band of pancreatic tissue that completely or partially surrounds the second duodenal portion, resulting in varying degrees of obstruction^(^^6^^)^. Its embryological origin begins between the fifth and seventh gestational weeks, when the two pancreatic buds (dorsal and ventral) rotate as part of the process of intestinal rotation^(^^6^^,^^7^^)^. During that period, the duodenum rotates from left to right, the ventral pancreatic bud typically migrates posteriorly and inferiorly, merging with the more caudal portion of the pancreatic head and the uncinate process, and the dorsal bud develops into the body and tail of the pancreas^(^^6^^)^. An annular pancreas is due to failure of the ventral bud to rotate, resulting in incarceration of the duodenum^(^^7^^)^. In general, an annular pancreas is symptomatic in children, especially in the neonatal period^(^^5^^)^, the main symptoms being bilious vomiting and abdominal distention^(^^6^^)^. In adults, it is typically asymptomatic and is diagnosed as an incidental finding^(^^5^^,^^8^^)^.

An abdominal X-ray of a patient with an annular pancreas will show the double-bubble sign, indicative of duodenal obstruction. Ultrasound, which is the first-line examination in the investigation of abdominal pain in children, reveals a fluid-distended duodenum and can identify the second duodenal portion incarcerated by pancreatic tissue. On computed tomography, pancreatic tissue surrounding the duodenum can also be seen^(^^9^^)^. In most cases, endoscopy is also performed. However, it should be borne in mind that even if the radiological and endoscopic findings both suggest an annular pancreas, the definitive diagnosis is established only during surgery. In patients with symptoms of obstruction, laparotomy can reveal a band of pancreatic tissue surrounding the second portion of the duodenum, supporting the diagnostic hypothesis, which can be confirmed by examining the resected specimen^(^^6^^)^.

## References

[1] Miranda CLVM, Sousa CSM, Cordão NGNP (2017). Intestinal perforation: an unusual complication of barium
enema. Radiol Bras.

[2] Pessôa FMC, Bittencourt LK, Melo ASA (2017). Ogilvie syndrome after use of vincristine: tomographic
findings. Radiol Bras.

[3] Niemeyer B, Correia RS, Salata TM (2017). Subcapsular splenic hematoma and spontaneous hemoperitoneum in a
cocaine user. Radiol Bras.

[4] Queiroz RM, Sampaio FDC, Marques PE (2018). Pylephlebitis and septic thrombosis of the inferior mesenteric
vein secondary to diverticulitis. Radiol Bras.

[5] Yigiter M, Yildiz A, Firinci B (2010). Annular pancreas in children: a decade of
experience. Eurasian J Med.

[6] Schmidt MK, Osvaldt AB, Fraga JCS (2004). Pâncreas anular - ressecção
pancreática ou derivação duodenal. Rev Assoc Med Bras.

[7] Sandrasegaran K, Patel A, Fogel EL (2009). Annular pancreas in adults. AJR Am J Roentgenol.

[8] Türkvatan A, Erden A, Türkoglu MA (2013). Congenital variants and anomalies of the pancreas and pancreatic
duct: imaging by magnetic resonance cholangiopancreaticography and
multidetector computed tomography. Korean J Radiol.

[9] Nijs E, Callahan MJ, Taylor GA (2005). Disorders of the pediatric pancreas: imaging
features. Pediatr Radiol.

